# Two Co(II) Isostructural Bifunctional MOFs via Mixed-Ligand Strategy: Syntheses, Crystal Structure, Photocatalytic Degradation of Dyes, and Electrocatalytic Water Oxidation

**DOI:** 10.3390/molecules29214989

**Published:** 2024-10-22

**Authors:** Siyu Yue, Mengqi Tuo, Yemeng Sheng, Xinyu Guo, Jiufu Lu, Dong Wang

**Affiliations:** 1 College of Chemical and Environment Science, Shaanxi University of Technology, Hanzhong 723001, China; 2 School of Medicine, Xizang Minzu University, Xianyang 712000, China

**Keywords:** solvothermal reactions, Co(II) organic framework, electrocatalytic properties, photocatalytic degradation

## Abstract

The solvothermal reactions involving cobalt ions with 5-methylisophthalic acid (H_2_MIP) and 1,3-bis(2-methylimidazol)propane (BMIP) yielded two cobalt(II) organic frameworks: {[Co_4_(MIP)_4_(BMIP)_3_]·1/2DMA}_n_ (**SNUT-31**) and {[Co_4_(MIP)_4_(BMIP)_3_]·(EtOH)_2_·H_2_O]}_n_ (**SNUT-32**) where DMA represents N,N-dimethylacetamide and EtOH signifies ethyl alcohol. Single-crystal X-ray diffraction analyses reveal that **SNUT-31** and **SNUT-32** possess an isomorphic structure, featuring a unique 2-fold interpenetration of 3D frameworks in a parallel manner. Notably, both **SNUT-31** and **SNUT-32** demonstrate remarkable performance in electrocatalytic oxygen evolution reactions and exhibit exceptional photocatalytic degradation capabilities against a model comprising three distinct dyes: rhodamine B, methyl orange, and methyl blue.

## 1. Introduction

As energy resources become increasingly limited, they have given rise to environmental concerns such as air pollution, energy waste, and greenhouse gas emissions [1]. As living standards rise and technology progresses, the search for alternative energy sources has become imperative. In this context, hydrogen production through electrocatalytic water splitting has emerged as a promising area of research [2,3]. Hydrogen is seen as a sustainable and eco-friendly energy alternative to fossil fuels, and its production method is cost-effective, relying solely on water and electricity [4]. This technology relies on two fundamental half-reactions: the Oxygen Evolution Reaction (OER) occurring at the anode and the Hydrogen Evolution Reaction (HER) at the cathode. The OER process [5], characterized by a complex four-electron transfer mechanism, is inherently sluggish, affecting energy conversion efficiency negatively and necessitating higher overpotentials, which, in turn, consume more energy [6]. Hence, there is a pressing need to develop efficient catalysts that can lower these overpotentials and accelerate the OER kinetics. Currently, Pt-group metals reign supreme as the most effective HER catalysts, while Ir- and Ru-based compounds are the benchmarks for OER catalysis. However, the focus of catalyst research has shifted towards creating low-cost, highly stable materials that maximize energy efficiency by minimizing the overpotential barrier [7]. To this end, significant efforts have been made to develop non-precious yet efficient electrocatalysts for water splitting. Among them, transition metal sulfides, carbides, and phosphides have shown promise [8,9,10,11,12], with cobalt-based compounds like CoS_2_ [13] and CoP [14], along with others [15,16,17,18], garnering significant interest for OER applications. Meanwhile, photocatalytic degradation of organic pollutants, as an effective technology, has the advantages of high efficiency, simplicity, and good reproducibility. For many years, people have been committed to developing low-cost, environmentally friendly, and highly stable photocatalysts [19,20,21,22,23,24,25], as well as Metal-Organic Framework (MOF) materials, with their versatility in composition, optics [26,27], gas adsorption/separation [28,29,30,31], photocatalysis [32], sensing [33,34,35], and drug transportation [36,37] due to their diverse compositions, varied structure types, and high porosity [38]. The dicarboxylic acid groups present in H_2_MIP ligands enable them to exhibit a wide range of coordination modes when paired with metal ions, rendering them well-suited for crafting functional complexes. Additionally, incorporating nitrogen-containing organic ligands into metal carboxylate systems tends to foster the creation of diverse topological structures more readily than relying solely on a single ligand.

In this work, by using 5-methylisophthalic acid (H_2_MIP) and 1,3-bis (2-methyl-imidazol) propane (BMIP) as the mixed ligands, and a two Cobalt(II) organic framework, {[Co_4_(MIP)_4_ (BMIP)_3_]·1/2DMA}_n_ (**SNUT-31**) and {[Co_4_(MIP)_4_(BMIP)_3_]·(EtOH)_2_·H_2_O]}_n_ (**SNUT-32**) have been synthesized and structurally characterized. Furthermore, the electrocatalytic and photocatalytic properties of **SNUT-31** and **SNUT-32** have been systematically investigated. The results showed that **SNUT-31** degraded methyl blue (MB) by 96.7% and **SNUT-32** degraded rhodamine B (Rh B) and methyl orange (MO) dyes by 77.4% and 80.5%, respectively. Meanwhile, **SNUT-32** had a C_dl_ value of 6.8 mF·cm^−2^ and a Tafel slope of 71 mV·dec^−1^, which showed high OER electrocatalytic activity and potential as an OER catalyst candidate.

## 2. Results and Discussion

### 2.1. Elemental Analyses

All the experimental results are consistent with the calculated values based on the formula given by X-ray single crystal diffraction, as given in the synthesis section.

### 2.2. Comment on IR, XRD and TGA

#### 2.2.1. FT-IR Spectra

**Figure 1:**
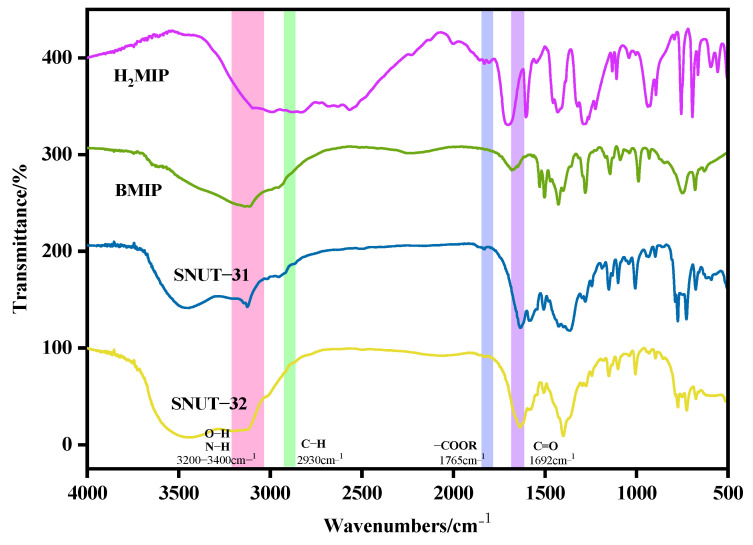
FTIR spectrum of SNUT-31 and SNUT-32.

In Figure 1, the infrared spectral analysis of both **SNUT-31** and **SNUT-32** covers a frequency span from 500 cm⁻^1^ to 4000 cm⁻^1^. Since both compounds incorporate identical ligands and metals, their infrared spectra exhibit comparable patterns stemming from the presence of similar functional groups. To streamline the analysis, we focused on **SNUT-31** as a representative sample. Within the infrared spectrum of **SNUT-31**, distinctive peaks are identified in the range of roughly 3000 cm⁻^1^ to 3200 cm⁻^1^, attributable to the -OH groups. Moreover, the peaks located at 1630 cm⁻^1^ signify the characteristic asymmetric and symmetric stretching vibrations of -C=O bonds. Lastly, the vibrational frequency range for the *ν*_C-N_ bond within the N-heterocycle of the ligand falls approximately between 640 cm⁻^1^ and 780 cm⁻^1^.

#### 2.2.2. Powder X-Ray Diffraction

The PXRD analysis conducted on **SNUT-31** and **SNUT-32** samples shows a precise correlation between the observed patterns and the simulated ones based on single crystal diffraction data (Figure S1), validating the purity of the synthesized products. Furthermore, to ensure that the intricate framework of the coordination polymers remained unperturbed after undergoing photocatalytic degradation of Rh B, MB, and MO dyes and exposure to a specific solution concentration, the samples were immersed in 0.3 mol/L solutions of NaOH, HNO_3_, and deionized water for 12 h. Subsequently, the soaked samples were vacuum-dried and subjected to heating at 100 °C for 3 h to activate them. The PXRD analysis of the activated samples confirmed that the XRD peak positions remained consistent with the simulated diffraction peaks, highlighting the exceptional stability of the framework structure in both **SNUT-31** and **SNUT-32** coordination polymers.

#### 2.2.3. Thermal Analysis

**Figure 2:**
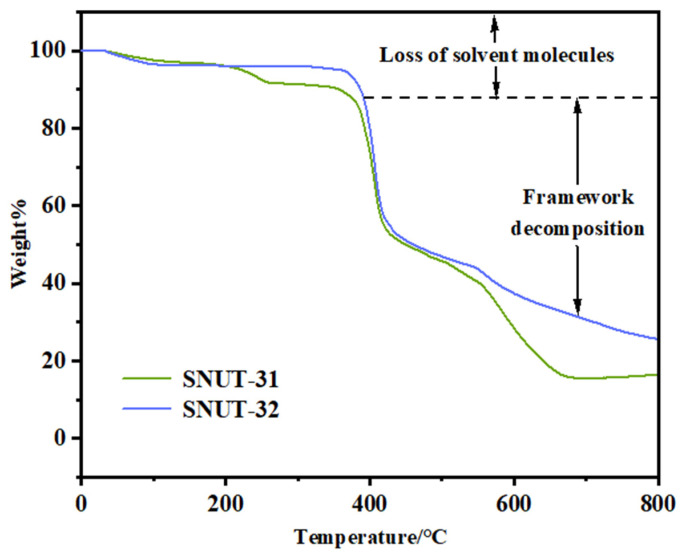
The TG curves of complexes SNUT-31 and SNUT-32.

During the Thermogravimetric (TG) analysis, performed in a nitrogen atmosphere from 25 to 800 °C with a heating rate of 10 °C per minute, both MOFs, **SNUT-31**, and **SNUT-32**, displayed comparable patterns of weight reduction, as evident in Figure 2. For SNUT-31, an initial weight loss of 3.7% (predicted at 4.8%) between 0 and 150 °C is associated with the elimination of 0.5 DMA molecules. Subsequently, a second weight loss phase, occurring from 300 to 550 °C, signifies the decomposition of ligands, resulting in the progressive degradation of the framework structure. Likewise, in the case of **SNUT-32**, an initial 3.7% weight loss (predicted at 4.5%) within 0 to 100 °C is attributed to the release of free water molecules. Subsequently, a notable weight loss is observed between 350 and 550 °C, indicative of the breakdown of the molecular structure.

### 2.3. Gas Adsorption

**Figure 3:**
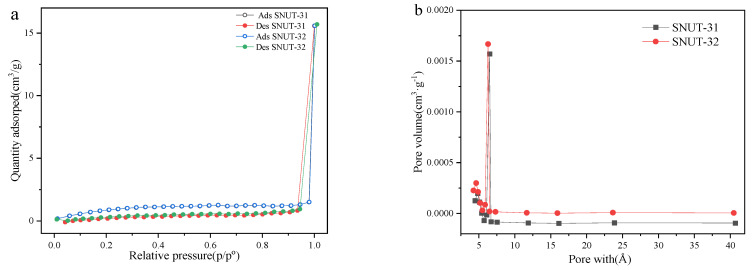
(**a**) N_2_ adsorption–desorption isotherms, and (**b**) corresponding pore size distribution curves of SNUT-31 and SNUT-32.

The gas adsorption properties of **SNUT-31** and **SNUT-32** were measured for N_2_ at 77 K. As shown in Figure 3a, both **SNUT-31** and **SNUT-32** show a typical type-IV isotherm corresponding to the saturated uptake of 15.6 cm^3^ g^−1^, 15.7 cm^3^ g^−1^ under 100 kPa, respectively. This confirms the microporous characteristic with 2.27, 2.29, and 5.87, 5.96 m^2^/g of the BET and Langmuir surface areas calculated, respectively (Figure 3a). In Figure 3b, the pore volume and pore size of **SNUT-31** and **SNUT-32** are 0.0016, and 0.0017 cm^3^ g^−1^, 0.65, and 0.63 nm, respectively, which agrees with the calculated pore size of 6 Å on the basis of crystal structure. Meanwhile, the similar S_bet_ and pore size further illustrates their isomorphic character.

### 2.4. Description of Crystal Structures

#### Crystal Structure of {[Co_4_(MIP)_4_(BMIP)_3_]·1/2DMA}_n_ (SNUT-31) and {[Co_4_(MIP)_4_(BMIP)_3_]·(EtOH)_2_·H_2_O]}_n_ (SNUT-32)

**Figure 4:**
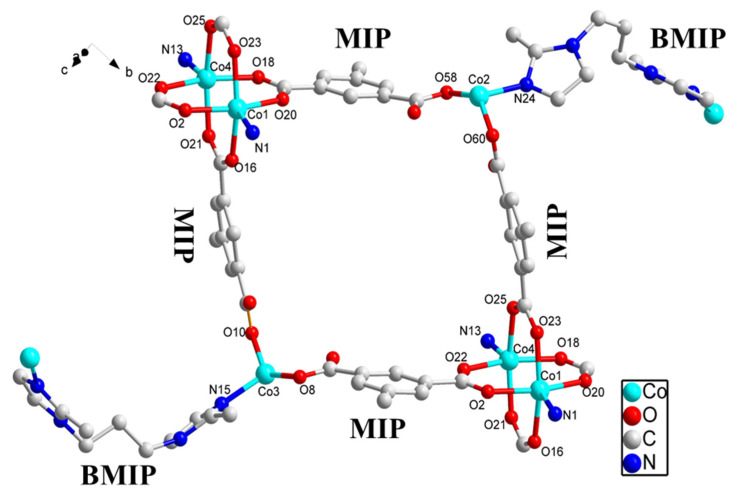
Coordination environments of Co(II) ion and the ligands in SNUT-31; hydrogen atoms are omitted for clarity.

**Figure 5:**
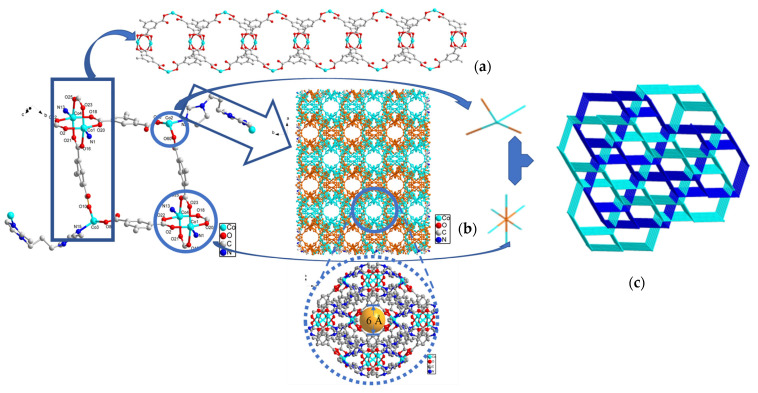
The compositions and corresponding structures of SNUT-31: (**a**) 1D chain, (**b**) View of 3D structure along the c axis in SNUT-31, (**c**) 2-fold 3D → 3D parallel interpenetrating structure of SNUT-31.

Through X-ray single crystal diffraction analysis, it was discovered that **SNUT-31** and **SNUT-32** crystallize within the monoclinic system, specifically under the space group *P2_1_/c* for **SNUT-31** and **SNUT-32**. Given the isostructural nature between **SNUT-31** and **SNUT-32**, the structural details of **SNUT-31** are thoroughly elaborated upon. The asymmetric unit of **SNUT-31** encompasses four Co(II) ions, four MIP^2−^ ions, and three BMIP ligands. Figure 4 showcases the two similar yet distinct coordination modes exhibited by the four Co(II) ions. Each Co1 ion forms a dual ring structure by coordinating with four carboxyl oxygen atoms from four MIP^2−^ anions and two nitrogen atoms from a single BMIP ligand. In contrast, each Co2 ion adopts a distorted tetrahedral geometry, resulting from its coordination with two carboxyl oxygen atoms from two MIP^2−^ anions and two nitrogen atoms from two BMIP ligands. The bond distances of Co-N and Co-O, ranging from 1.920(7) to 2.257(8) Å and 1.966(4) to 2.058(4) Å, respectively, suggest a distorted octahedral coordination environment. Furthermore, adjacent Co1 and Co2 ions connect via oxygen atoms in MIP^2−^ anions, forming 1*D* cyclic chains (as seen in Figure 5a). These chains subsequently serve as connectors between numerous parallel chains, bridging through the two unique coordination modes to assemble a porous 3*D* framework (depicted in Figure 5b), in which exists a hole with radius of 6 Å, from the angle of the c-axis. Lastly, Figure 5c reveals that the individual 3*D* frameworks interpenetrate due to the vast void space, yielding a complex 2-fold 3*D* → 3*D* interpenetrating structure.

### 2.5. Electrochemical Performance

**Figure 6:**
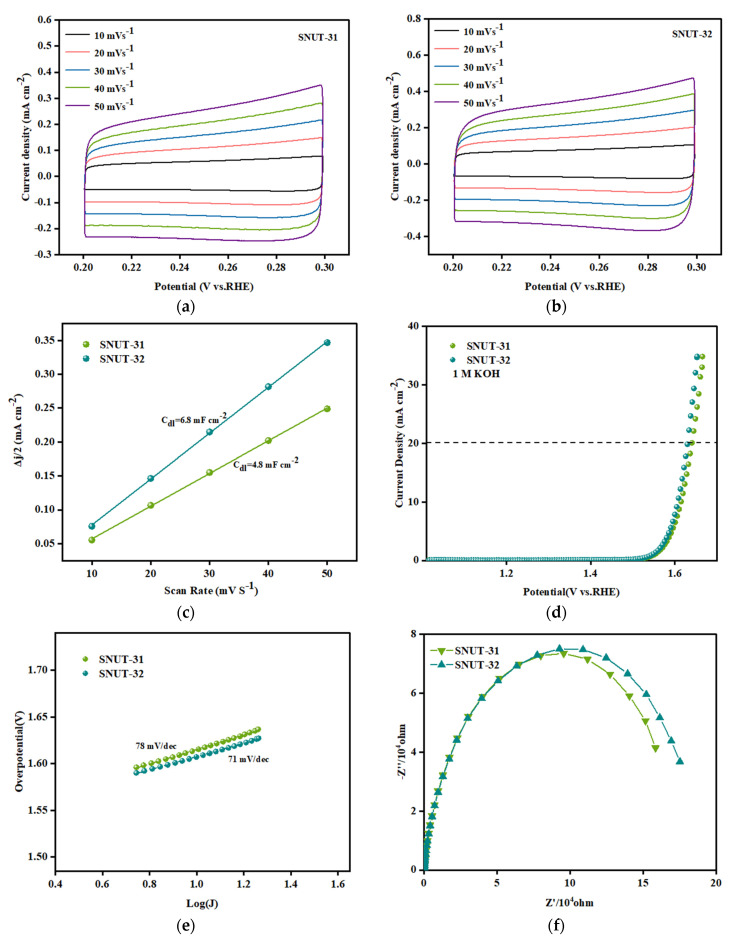
CV curves of SNUT-31 (**a**) and SNUT-32 (**b**) at different scanning rates of 10~50mV·s^−1^; Double-layer capacitance (C_dl_) (**c**) of SNUT-31 and SNUT-32 within 0.2~0.3 V; LSV polarization curve (**d**) Tafel slope (**e**) and electrochemical impedance diagram (**f**) of SNUT-31 and SNUT-32 corresponding to OER in 1 M KOH electrolyte at a scanning rate of 5 mV·s^−1^.

In the setup of a three-electrode configuration featuring a glassy carbon electrode, a carbon rod, and a saturated calomel electrode, a cyclic voltammetry (CV) experiment was performed with a scanning rate of 5 mV/s, utilizing a 1 mol/L KOH solution as the electrolytic medium. Under identical conditions, the electrocatalytic performance of **SNUT-31** and **SNUT-32** was scrutinized. The electrochemical surface area (ECSA) for both **SNUT-31** and **SNUT-32** was derived from the conducted CVs, as depicted in Figure 6a,b. Additionally, the double-layer capacitance (C_dl_) was computed by analyzing the fluctuations in current density within the 0.2 to 0.3 V range across varying scanning rates, illustrated in Figure 6c. The findings revealed that **SNUT-32** boasts a higher C_dl_ value of 6.8 mF·cm^−2^ compared to **SNUT-31**’s 4.8 mF·cm^−2^, implying that **SNUT-32** exhibits superior OER catalytic activity. This enhanced activity facilitates a larger interface area between the electrode and electrolyte, thereby exposing a greater number of active sites. When evaluating catalyst activity, a pivotal metric is the overpotential achieved at a current density of 20 mA·cm^−2^. As evident from Figure 6d, **SNUT-32** demonstrates a lower overpotential of 400 mV compared to **SNUT-31**’s 410 mV, conclusively indicating that **SNUT-32** possesses a higher catalytic efficiency.

To gain further insights into the kinetics of the OER reaction, the Tafel slope was calculated from the polarization curve utilizing the Tafel equation (η = b log j + a), as shown in Figure 6e. Notably, **SNUT-32**, with a Tafel slope of 71 mV·dec^−1^, demonstrates a lower slope compared to **SNUT-31** (78 mV·dec^−1^), suggesting that **SNUT-32** facilitates a more rapid kinetic reaction during catalysis than **SNUT-31**.

To investigate the electron transfer kinetics within the OER process, we conducted electrochemical impedance spectroscopy (EIS). The results, presented in Figure 6f, reveal that **SNUT-31** exhibits a smaller arc radius in the Nyquist plot’s high-frequency region. This observation indicates that **SNUT-31** possesses a lower charge transfer resistance (Rct) compared to **SNUT-32**, leading to a faster charge transfer rate at the electrode–electrolyte interface. Consequently, **SNUT-31** displays superior OER electrocatalytic performance, confirming that it exhibits accelerated electron transfer kinetics during the OER process. As depicted in Figure S3a,b, the calculated TOFs of **SNUT-31** is slightly higher than that of **SNUT-32**. For example, the TOF values of **SNUT-31** and **SNUT-32** are 0.00214 and 0.00157 s^−1^ at the overpotential of 500 mV, respectively, based on the active center. These results confirm the better electrocatalytic activity of **SNUT-31** for OERs than that of **SNUT-32**.

### 2.6. Photocatalytic Degradation

**Figure 7:**
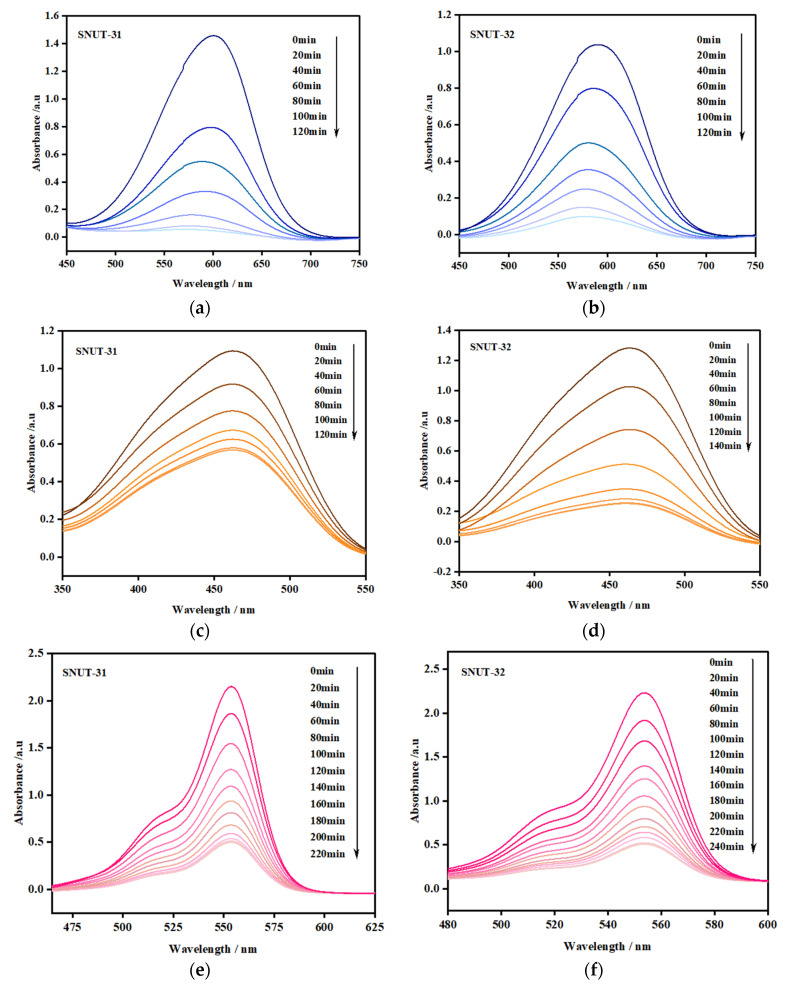
(**a**,**c**,**e**) Absorption spectra of MB, MO, and Rh B solutions during photocatalytic degradation by SNUT-31, (**b**,**d**,**f**) absorption spectra of MB, MO, and Rh B solutions during photocatalytic degradation by SNUT-32.

The experimental results depicted in Figure 7 demonstrate that as the irradiation time increased, the intensities of the MB, MO, and Rh B bands diminished noticeably in the presence of both **SNUT-31** and **SNUT-32**. Specifically, for **SNUT-31**, Figure 7a illustrates how the concentrations of MB, Rh B, and MO(C) fluctuated with the reaction time (t), revealing degradation efficiencies of roughly 96.7%, 76.9%, and 48.1%, respectively, after 120, 220, and 120 min. Analogously, for **SNUT-32**, Figure 7b shows similar variations in concentrations with time, yielding degradation rates approaching 91%, 77.4%, and 80.5% after 120, 240, and 140 min, respectively.

**Figure 8:**
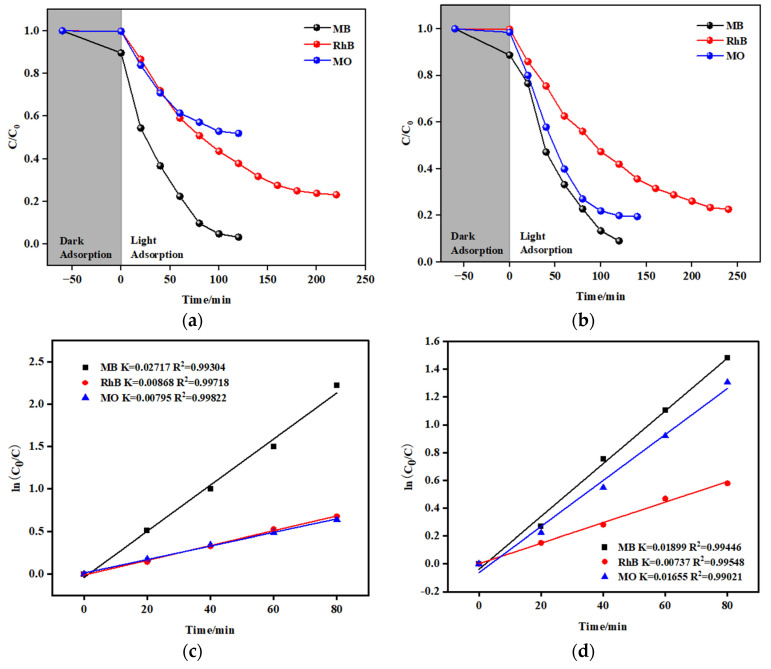
The changes in the degradation rate (C/C_0_) of SNUT-31 (**a**) and SNUT-32 (**b**) with light exposure time, linear logarithm of degradation rate of SNUT-31 (**c**) and SNUT-32 (**d**) with light exposure time.

**Figure 9:**
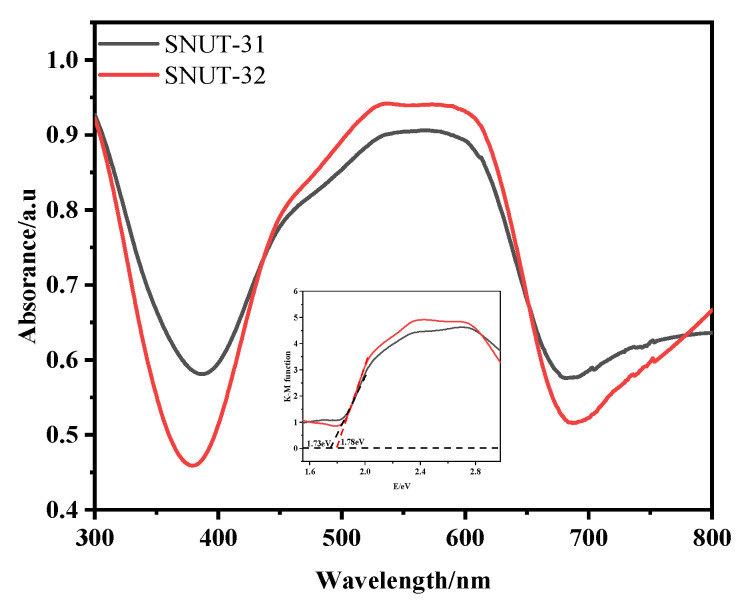
UV–vis absorption spectra and corresponding band gap diagrams of SNUT-31 and SNUT-32 (converted from Kubelka–Munk).

**Figure 10:**
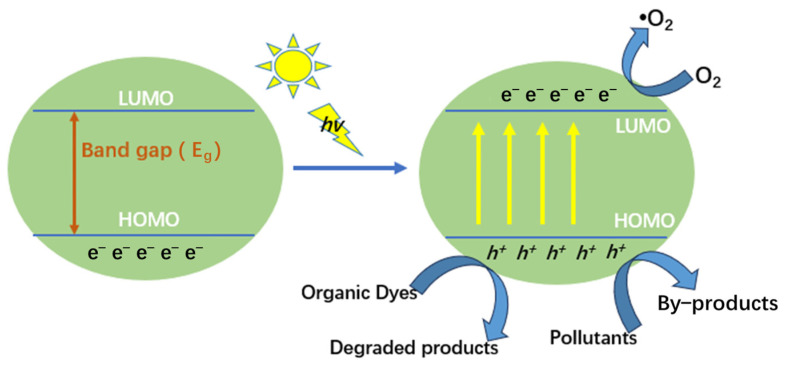
Electro transfer between HOMO and LUMO energy levels, production reactive species and photodegradation of organic dyes.

Upon comparing the constant degradation rates (k) for both **SNUT-31** (Figure 7c) and **SNUT-32** (Figure 7d), it becomes clear that methyl blue (MB) consistently displayed the highest photocatalytic degradation rate. For context, a blank control experiment was conducted to assess the degradation rates of the three organic dyes in the absence of the coordination polymer and mercury lamp irradiation. As evidenced in Figure 8a–d, while irradiation from the mercury lamp alone partially accelerated the degradation process, the combination of both the lamp and the coordination polymer significantly boosted the degradation rates. These findings highlight the remarkable photocatalytic capabilities of **SNUT-31** and **SNUT-32**, particularly towards methyl blue (MB), among cationic dyes. However, both **SNUT-31** and **SNUT-32** show high stability in this reaction. Five iterations of the cycle (Figure S4a,b) revealed no obvious decrease of degradation efficiency of methyl blue, which can still reached 94.1 and 89.4, respectively. The used **SNUT-31** and **SNUT-32** retained its structure after the reaction (Figure S1). According to the reported mechanism of Co-based MOFs in the degradation of organic dyes, the ability of photocatalysts to capture light plays a key role [23,39,40]. The optical activity of **SNUT-31** and **SNUT-32** were investigated using the UV-vis diffuse reflectance spectrum (DRS), from which the band gap (Eg) of **SNUT-31** and **SNUT-32** can be calculated as 1.73 eV and 1.78 eV from the Tauc plot (Figure 9), indicating that **SNUT-31** and **SNUT-32** have intense absorption within the visible light range. As shown in Figure 10, the general process of photocatalytic MOF is the transfer of electrons from the highest occupied molecular orbital (HOMO) to the lowest occupied molecular orbital (LUMO) by the ligand. The electrons are transferred from the highest occupied molecular orbital (HOMO) to the lowest unoccupied molecular orbital (LUMO), resulting in the formation of an excited state. In the excited state, electrons are transferred between the metal center and the ligand while active species are produced, resulting in photodegradation of organic dyes. Due to the isomeric nature of SNUT-31 and SNUT-32, as well as the similar properties, the generation of reactive oxidizing species by SNUT-31 under UV light irradiation was investigated via the DMPO spin-trapping electron paramagnetic resonance (EPR) technique to verify the ability of active species to influence photodegradation. The results are shown in Figure S5. Notably, no reactive oxidizing species were detected in the dark condition. However, after 5 min of visible light irradiation, ·O_2_^−^ species was clearly identified. This observation unequivocally establishes ·O_2_^−^ as the reactive oxidizing species operating during the degradation process under UV irradiation.

## 3. Experimental

### 3.1. Materials and Methods

All used chemical reagents are available from Shanghai Aladdin Biochemical Technology Co., Ltd. (Shanghai, China). Crystal data were obtained on a Bruker APEX-II CCD X-diffractometer (Bruker, Billerica, MA, USA). X-ray powder diffraction patterns were obtained on a Bruker D8 ADVANCE (Bruker). IR spectra were recorded as KBr pellets on a Thermo scientific Nicolet™ iS20 FT-IR spectrometer (Thermo Scientific, Waltham, MA, USA). Electrochemical investigation data were obtained on a Shanghai Chenhua CHI660E workstation (Shanghai, China).

### 3.2. Synthesis of {[Co_4_(MIP)_4_(BMIP)_3_]·1/2DMA}_n_ (SNUT-31)

A mixture of Co(NO_3_)_2_·6H_2_O (0.15 mmol), BMIP (0.10 mmol), H_2_MIP (0.10 mmol), HNO_3_ (0.5 mmol), and DMA (3 mL) was placed in a vial, heated to 110 °C for 3 d, and then cooled to room temperature over 24 h. Purplish red crystals of **SNUT-31** were collected, washed with water, and air-dried (Yield: 78%). Elemental analysis (wt%) was calculated for C_137_H_142_Co_8_N_24_O_32_ (*Mr* = 3108.18): C 52.93, H 4.57, N 10.82; the following was found: C 52.88, H 4.49, N 11.21. IR (cm^–1^): 3126(s), 2951(w), 1633(s), 1583(w), 1507(s), 1306(s), 1007(m), and 774(m).

### 3.3. Synthesis of {[Co_4_(MIP)_4_(BMIP)_3_]·(EtOH)_2_·H_2_O]}_n_ (SNUT-32)

The same procedure as that for **SNUT-31** was used except that DMA was replaced by EtOH. Purplish red crystals of **SNUT-32** were collected, washed with water, and air-dried (Yield: 80%). Elemental analysis (wt %) was calculated for C_73_H_86_Co_4_N_12_O_19_ (*Mr* = 1671.25): C 52.42, H 5.15, N 10.05; the following was found: C 52.38, H 5.09, N 11.12. IR (cm^–1^): 3123(v), 3022(v), 1636(s), 1506(s), 1400(s), 1150(m), 1005(w), and 773(m).

### 3.4. Electrocatalytic Properties

To prepare the electrode, mix **SNUT-31** or **SNUT-32** (10 mg), H_2_O (500 μL), EtOH (490 μL), and Nafion (10 μL). Perform ultrasonic dispersion for 20 min, then drop the resulting suspension onto the surface of the working electrode and allow it to air-dry. This method will adhere **SNUT-31** or **SNUT-32** to the working electrode. Subsequently, use a carbon rod as the counter electrode, a saturated calomel electrode (SCE) as the reference electrode, and KOH (1 mol/L) as the electrolyte. Analyze the OER properties of the coordination polymer using cyclic voltammetry (CV) and linear sweep voltammetry (LSV). Additionally, obtain the material’s electron transfer ability through electrochemical impedance spectroscopy (EIS).

### 3.5. Solid-State Uv-Vis Absorption

To assess the light absorption capabilities of **SNUT-31** and **SNUT-32**, UV–vis diffuse reflectance spectra (DRSs) were acquired. Figure 5 reveals that both compounds display wide absorption bands, hinting at their potential to enhance photocatalytic degradation within the ultraviolet spectrum. Subsequently, the band gap energy (Eg) of **SNUT-31** and **SNUT-32** was derived using the Kubelka–Munk plot. The *E_g_* values were found to be 1.73 eV for **SNUT-31** and 1.78 eV for **SNUT-32**, indicating their suitability as photocatalysts in the ultraviolet region. To validate this, the photocatalytic efficacy of the synthesized **SNUT-31** and **SNUT-32** was tested by examining their ability to decompose aqueous solutions of methyl blue (MB), rhodamine B (RhB), and methyl orange (MO).

### 3.6. Determination of Crystal Structures

Dimensions of suitable size were selected for the above three crystals for data collection, which was performed at 293(2) K with a CCD four-circle diffractometer XtaLAB Synergy, Dualflex, HyPix, with mirror monochromated Cu Ka radiation (λ = 1.54184 Å). The crystal structure was solved by direct methods with the SHELXT 2014/5 (Sheldrick, 2014) and refined with the SHELXL 2018/3 (Sheldrick, 2015) [41,42]. Crystallographic data and experimental details of structural analyses for molecular are summarized in Table S1. The main bond lengths and angles of **SNUT-31** and **SNUT-32** are listed in Table S2.

## 4. Conclusions

In summary, we achieved the synthesis of two cobalt(II) organic frameworks, **SNUT-31** and **SNUT-32**, through solvothermal reactions involving the ligands H_2_MIP and BMIP. Two MOFs, specifically {[Co_4_(MIP)_4_(BMIP)_3_]·1/2DMA}_n_ (**SNUT-31**) and [Co_4_(MIP)_4_(BMIP)_3_ (EtOH)_2_H_2_O]_n_ (**SNUT-32**), with DMA representing *N*,*N*-dimethylacetamide and EtOH signifying ethyl alcohol, are isomorphic, which features 2-fold 3D → 3D parallel interpenetrating frameworks. Notably, **SNUT-32** exhibits remarkable catalytic prowess in promoting the oxygen evolution reaction (OER) under electrochemical conditions. Moreover, both **SNUT-31** and **SNUT-32** show exceptional photocatalytic degradation efficiency when applied to a model system containing three dyes: rhodamine B (Rh B), methyl orange (MO), and methyl blue (MB). In addition, this work is comparable to some previously reported MOF-based catalysts of OER performance, as shown in Table 1, compared to the photocatalytic degradation of MOF based on MB, MO, and Rh B, as shown in Table 2.

Crystallographic data for the structural analysis have been deposited with the Cambridge Crystallographic Data Center: 2368802 for **SNUT-31**, 2368803 for **SNUT-32**. Copies of the data can be obtained free of charge on application to the Director, CCDC, 12 Union Road, Cambridge, CB2 1EZ, UK (Fax: +44-1223-336033; e-mail: deposit@ccdc.cam.ac.uk or http://www.ccdc.cam.ac.uk accessed on 20 October 2023).

## Figures and Tables

**Table 1:** The OER properties of some catalysts are compared.

MOF	Current Density (mA cm^−2^)	Overpoten Tial (mV)	Tafel Slope (mV dec^–1^)	Ref.
Fe-MOF/FF	100	371	114.2	[43]
Ni-MOF/FF	100	383	160.7	[43]
NiFe-MOF/FF	100	240	73.4	[43]
Ag@Co-MOF	10	344	107	[44]
CoNi MOF-mCNTs	10	306	42	[39]
FeNiCo-MOF	10	239	42.4	[45]
CoNiMn-MOF	20	220	66	[40]
CC/MOF-CoSe_2_@MoSe_2_	20	211.4	96.61	[46]

**Table 2:** Photocatalytic degradation performance of functionalized MOFs for organic dyes.

MOF	Dye	Time (min)	Degradation Efficiency (%)	Ref.
HPU-4@AgBr	MB	60	95	[19]
MIL-88B(Fe)@BiOI	MB	80	80	[20]
UiO-66@a-Fe_2_O_3_	MB	55	100	[21]
TCPP-La	MO	125	16	[22]
[Co(H_3_tpb)(Hbtc)]_n_	MO	150	75.3	[23]
Ag@MOF-525	RhB	60	91	[24]
MIL−88B(Fe)	RhB	120	96	[25]

## Data Availability

The data presented in this study are available in the article.

## Supplementary Materials

The following supporting information can be downloaded at: https://www.mdpi.com/article/10.3390/molecules29214989/s1, Table S1. Crystal data of SNUT-31~SNUT-32. Table S2. Selected bond lengths (Å) and angles (◦) for SNUT-31 and SNUT-32. Figure S1. The powder XRD patterns of (a) SNUT-31 and (b) SNUT-32. Figure S2. The blank experiment of MB (a,b), MO (c,d), and Rh B (e,f) solutions during photocatalytic degradation by SNUT-31 and SNUT-32. Figure S3. a. The turnover frequency (TOF) of SNUT-31 at different potentials. b. The turnover frequency (TOF) of SNUT-32 at different potentials. Figure S4. a. Reusability of SNUT-31 for photocatalytic degradation of MB. b. Reusability of SNUT-32 for photocatalytic degradation of MB. Figure S5. EPR spectra of DMPO-⋅O_2_^−^ with SNUT-31.
