# Supplementary material for: Two Co(II) Isostructural Bifunctional MOFs via Mixed-Ligand Strategy: Syntheses, Crystal Structure, Photocatalytic Degradation of Dyes, and Electrocatalytic Water Oxidation
Source: Molecules. 2024 Oct 22;29(21):4989. doi: 10.3390/molecules29214989 (PMC12120997; doi:10.3390/molecules29214989)
Supplement: Supplementary file 1 [file molecules-29-04989-s001.zip › molecules-3246888-supplementary.pdf]

Support information

Two Co(II) Isostructural Bifunctional MOFs via Mixed-Ligand Strategy: Syntheses, Crystal Structure, Photocatalytic Degradation of Dyes, and Electrocatalytic Water Oxidation

Siyu Yue [1], Mengqi Tuo [1], Yemeng Sheng [2], Xinyu Guo [1], Jiufu Lu [1,*] and Dong Wang [2,*]

[1] College of Chemical and Environment Science, Shaanxi University of Technology, Hanzhong 723001, China
[2] School of Medicine, Xizang Minzu University, Xianyang 712000, China
* Correspondence: jiufulu@snut.edu.cn (J.L.); dwang1987@126.com (D.W.); Tel./Fax: +86-09162641660 (J.L.)

Table S1. Crystal data of SNUT-31~SNUT-32.

Compounds	SNUT-31	SNUT-32
Formula	$C_{137}H_{142}Co_8N_{24}O_{32}$	$C_{73}H_{86}Co_4N_{12}O_{19}$
Fw	3108.18	1671.25
T/K	293(2)	293(2)
Crystal system	Monoclinic	Monoclinic
Space group	*P2₁/c*	*P2₁/c*
a/Å	18.8317(6)	18.7452(4)
b/Å	15.0445(4)	13.5066(2)
c/Å	28.6521(12)	31.8498(4)
α/°	90.00	90.00
β/°	100.466(4)	95.4300(10)
γ/°	90.00	90.00
V/Å^3	7982.5(5)	8027.7(2)
Z	2	4
Dc/g · cm^{-3}	1.293	1.383
F(000)	3208	3472
Index ranges	-22 ≤ h ≤ 22, -16 ≤ k ≤ 17, -34 ≤ l ≤ 34	-22 ≤ h ≤ 22, -14≤ k ≤ 16, -33 ≤ l ≤ 37
Reflection/unique	57948/14217	68961/13899
R_1, wR_2 [$I > 2(I)$]	0.0782,0.2319	0.0678,0.1323
R_1, wR_2 (all data)	0.1141,0.2641	0.0884,0.1463

$R_1 = \sum(||F_o|-|F_c||)/\sum|F_o|$, $wR_2 = [\sum(||Fo|^2-|Fc||^2)^2/\sum w(F_o^2)]^{1/2}$

Table.S2. Selected bond lengths (Å) and angles (◦) for SNUT-31 and SNUT-32

**SNUT-31**			
Co(1)-Co(4)	2.8439(8)	O(2)#1-Co(1)-Co(4)	82.36(11)

Co(1)-O(2)#1	2.034(4)	O(2)#1-Co(1)-O(20)	163.13(15)
Co(1)-O(20)	2.036(4)	O(2)#1-Co(1)-O(23)#1	85.90(17)
Co(1)-O(23)#1	2.058(4)	O(2)#1-Co(1)-N(1)#2	90.1(3)
Co(1)-O(16)	2.021(4)	O(20)-Co(1)-Co(4)	80.85(11)
Co(1)-N(1)#2	2.057(9)	O(20)-Co(1)-O(23)#1	88.47(17)
Co(1)-N(12)#2	2.008(7)	O(20)-Co(1)-N(1)#2	106.7(3)
Co(4)-O(22)#1	2.027(4)	O(23)#1-Co(1)-Co(4)	75.89(15)
Co(1)-Co(4)	2.8439(8)	O(2)#1-Co(1)-Co(4)	82.36(11)
Co(1)-O(2)#1	2.034(4)	O(2)#1-Co(1)-O(20)	163.13(15)
Symmetry code: #1 (+X, -1+Y, +Z) #2 (1/2-X, +Y, 3/2-Z) #3 (1/2-X, -1+Y, 1/2-Z)			
**SNUT-32**			
Co(1)-Co(2)	2.8387(9)	N(2)-Co(1)-Co(2)	169.05(11)
Co(1)-N(2)	2.037(4)	O(1)-Co(1)-Co(2)	84.84(11)
Co(1)-O(1)	2.018(4)	O(1)-Co(1)-N(2)	105.09(15)
Co(1)-O(5)	2.016(3)	O(5)-Co(1)-Co(2)	81.10(10)
Co(2)-N(6)	2.027(4)	N(6)-Co(2)-Co(1)	168.62(14)
Co(2)-O(2)	2.045(3)	O(2)-Co(2)-Co(1)	78.07(11)
Co(2)-O(6)	2.047(3)	O(2)-Co(2)-N(6)	91.84(17)
Co(2)-O(10)	2.031(3)	O(6)-Co(2)-N(6)	92.79(16)
Symmetry code: #1(+X, -1/2-Y, 1/2+Z)			

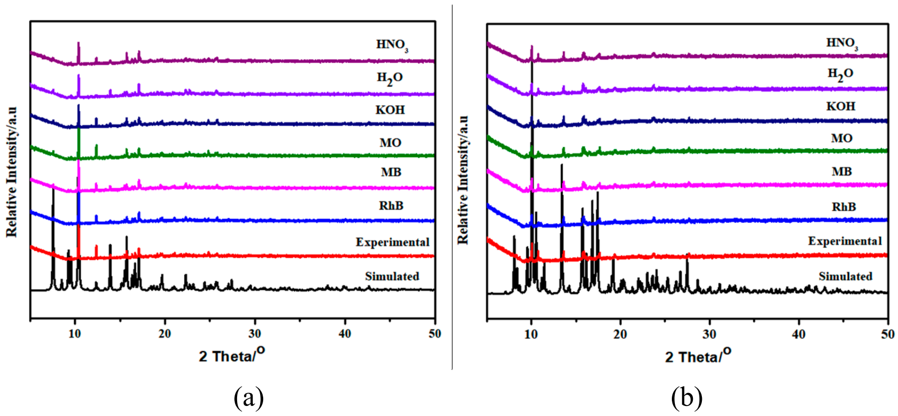

Figure S1. The powder XRD patterns of (a)SNUT-31 and (b)SNUT-32.

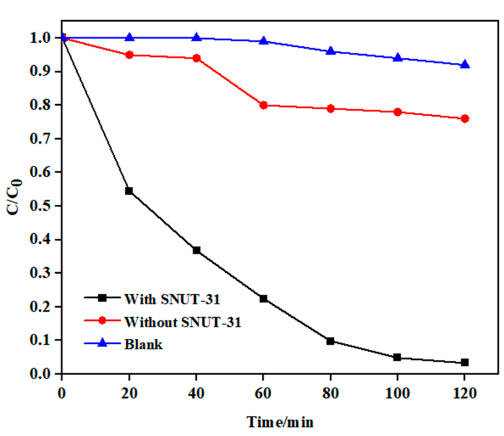

(a)

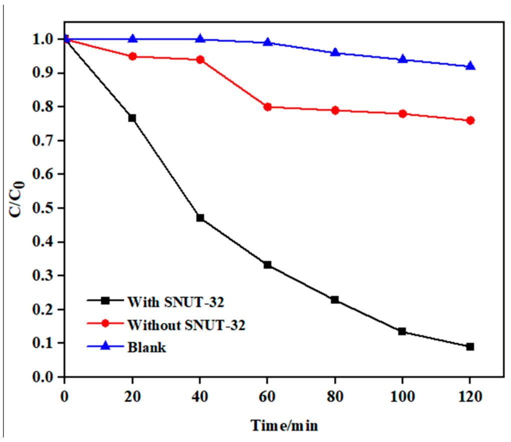

(b)

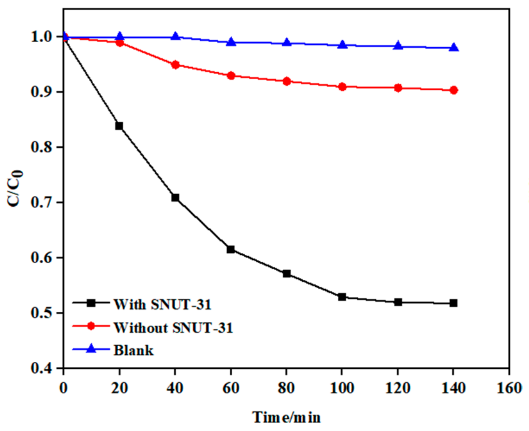

(c)

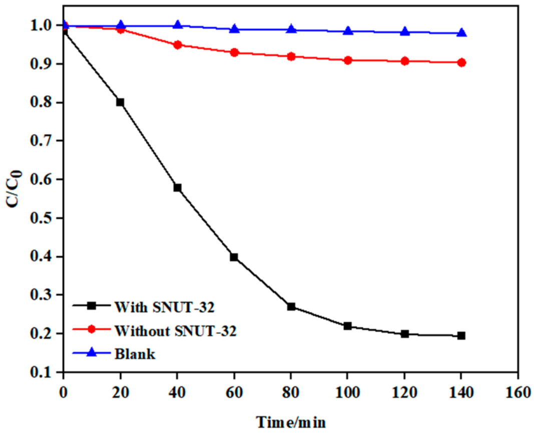

(d)

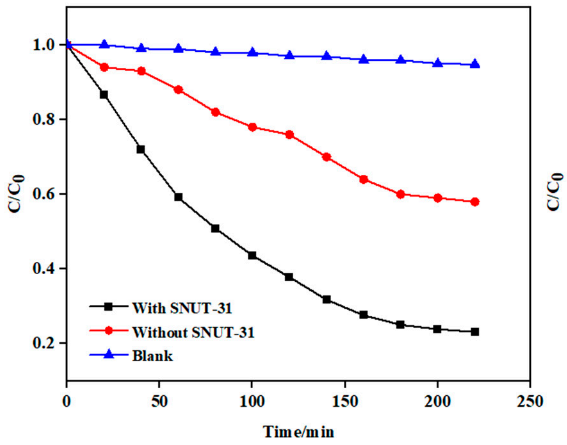

(e)

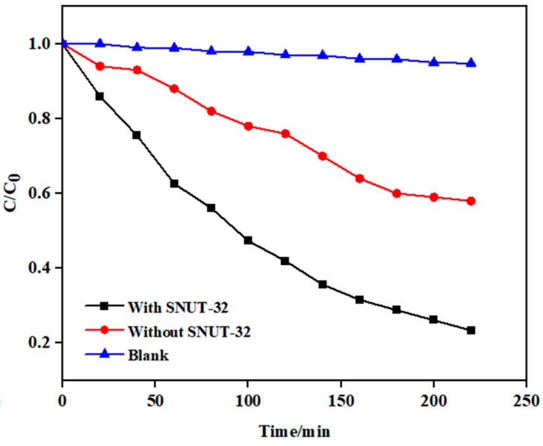

(f)

Figure S2. The blank experiment of MB(a) (b), MO(c) (d), and Rh B(e) (f) solutions during photocatalytic degradation by SNUT-31 and SNUT-32.

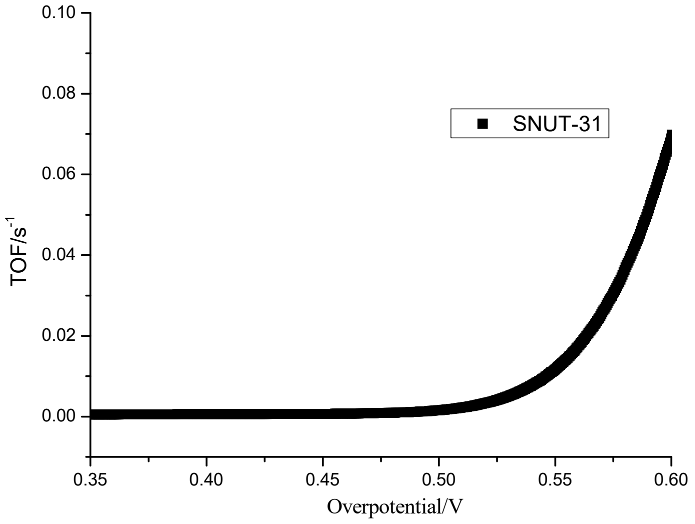

Figure S3a. The turnover frequency (TOF) of SNUT-31 at different potentials.

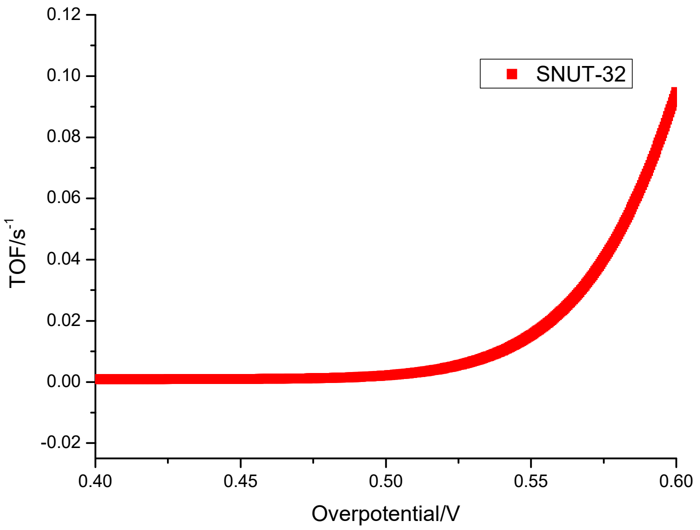

Figure S3b. The turnover frequency (TOF) of SNUT-32 at different potentials

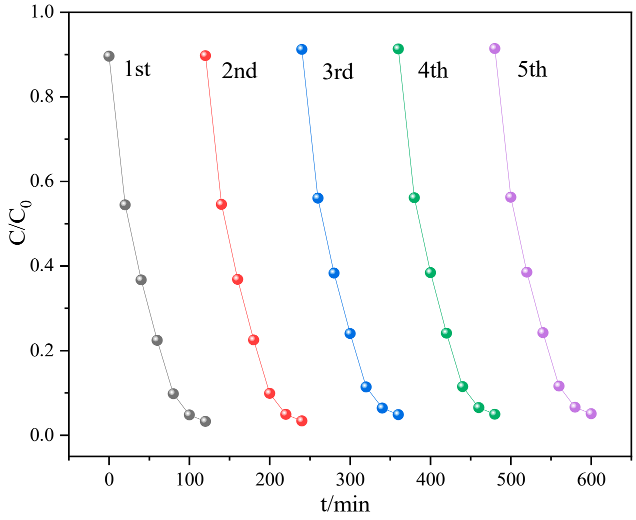

Figure S4a. Reusability of SNUT-31 for photocatalytic degradation of MB

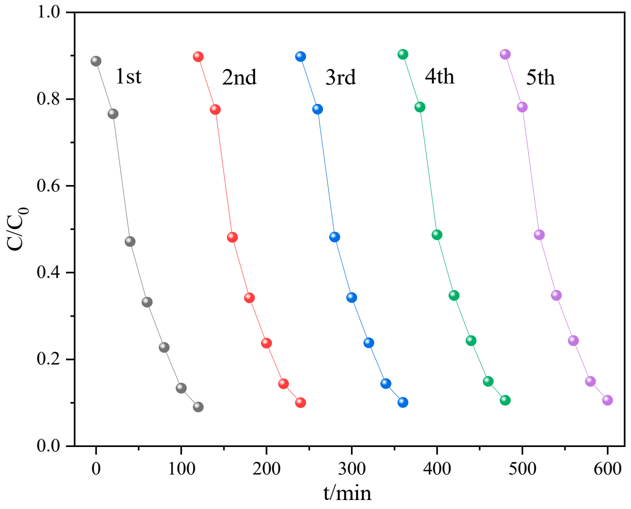

Figure S4b. Reusability of SNUT-32 for photocatalytic degradation of MB

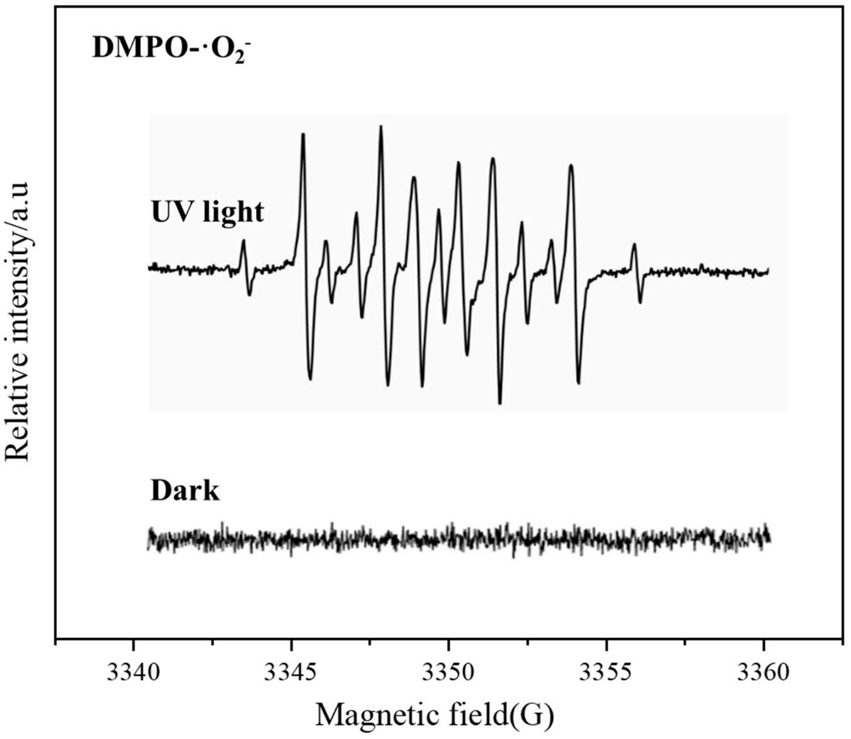

Figure S5. EPR spectra of DMPO-·O_2^- with SNUT-31.
